# An Autopsy Study Describing Causes of Death and Comparing Clinico-Pathological Findings among Hospitalized Patients in Kampala, Uganda

**DOI:** 10.1371/journal.pone.0033685

**Published:** 2012-03-14

**Authors:** Janneke A. Cox, Robert L. Lukande, Ann M. Nelson, Harriet Mayanja-Kizza, Robert Colebunders, Eric Van Marck, Yukari C. Manabe

**Affiliations:** 1 Department of Clinical Sciences, Institute of Tropical Medicine, Antwerp, Belgium; 2 Department of Pathology, Makerere University, Kampala, Uganda; 3 Joint Pathology Center, Silver Spring, Maryland, United States of America; 4 Department of Internal Medicine, Makerere University, Kampala, Uganda; 5 Faculty of Medicine, University of Antwerp, Antwerp, Belgium; 6 Infectious Diseases Institute, Makerere University College of Health Sciences, Kampala, Uganda; 7 Division of Infectious Diseases, Department of Medicine, Johns Hopkins University School of Medicine, Baltimore, Maryland, United States of America; Johns Hopkins Bloomberg School of Public Health, United States of America

## Abstract

**Background:**

Information on causes of death in HIV-infected patients in Sub-Saharan Africa is mainly derived from observational cohort and verbal autopsy studies. Autopsy is the gold standard to ascertain cause of death. We conducted an autopsy study to describe and compare the clinical and autopsy causes of death and contributory findings in hospitalized HIV-infected and HIV-uninfected patients in Uganda.

**Methods:**

Between May and September 2009 a complete autopsy was performed on patients that died on a combined infectious diseases gastroenterology ward in Mulago Hospital in Kampala, Uganda. Autopsy cause of death and contributing findings were based on the macro- and microscopic post-mortem findings combined with clinical information. Clinical diagnoses were reported by the ward doctor and classified as confirmed, highly suspected, considered or not considered, based on information derived from the medical chart. Results are reported according to HIV serostatus.

**Results:**

Fifty-three complete autopsies were performed in 66% HIV-positive, 21% HIV-negative and 13% patients with an unknown HIV serological status. Infectious diseases caused death in 83% of HIV-positive patients, with disseminated TB as the main diagnosis causing 37% of deaths. The spectrum of illness and causes of death were substantially different between HIV-positive and HIV-negative patients. In HIV-positive patients 12% of postmortem diagnoses were clinically confirmed, 27% highly suspected, 16% considered and 45% not considered. In HIV-negative patients 17% of postmortem diagnoses were clinically highly suspected, 42% considered and 42% not considered.

**Conclusion:**

Autopsy examination remains an important tool to ascertain causes of death particularly in settings with limited access to diagnostic testing during life. HIV-positive patients continue to die from treatable and clinically undiagnosed infectious diseases. Until rapid-point of care testing is available to confirm common infections, empiric treatment should be further investigated.

## Introduction

Although the roll-out of antiretroviral therapy (ART) in 2004 has decreased mortality among the HIV-infected, HIV mortality rates in Sub-Saharan Africa remain high. Globally, an estimated 1.8 million HIV/AIDS-related deaths occurred in 2009, with 72% of the deaths in Africa (1.3–1.8 million) [1]. In Uganda, an estimated 1.1 million people were HIV-infected and 61.000 deaths were caused by HIV-related illness in 2008 [2]. Due to limited diagnostic testing in most sub-Saharan African countries, the exact cause of death is often difficult to ascertain. Even in developed countries, discrepancies between clinical and autopsy diagnoses have been reported to be as high as 50% [3], [4]. Autopsy remains the gold standard for confirming the cause of death. Previous studies have shown that (partial) autopsy is a feasible procedure in Sub-Saharan Africa, both in study settings and as a general practice [5], [6], [7].

The autopsy studies performed on HIV-infected patients in Sub-Saharan Africa have highlighted the central contribution of opportunistic infectious diseases to mortality [8]. These studies did not include any patients on antiretroviral therapy (ART) and only a limited number of patients taking cotrimoxazole prophylaxis. Information on causes of death in HIV-infected patients on ART in Sub-Saharan Africa is scarce and is derived exclusively from observational and verbal autopsy studies [9], [10], [11].

We conducted a prospective autopsy study among individuals dying on a combined infectious diseases gastroenterology ward of Mulago National Referral Hospital in Kampala, Uganda. We sought to describe and compare the clinical and autopsy causes of death and contributory findings in HIV-positive and HIV-negative patients.

## Methods

### Study setting

Mulago National Tertiary Referral Hospital is located in Kampala, Uganda and is a university teaching centre. Over the last 10 years, approximately 6800 patients died annually in Mulago Hospital including maternal and child deaths. Based on data from the mortuary, the autopsy rate in Mulago Hospital over the past decade has been stable at 5%. This study was conducted on a combined infectious diseases and gastroenterology ward. The study team included clinical doctors and pathologists.

### Study design

Next of kin of all patients that died on weekdays in the period from May–September 2009 on the study ward were asked for written informed consent to participate in the study. Both verbal and written information about the research and the autopsy procedure was provided in English and Luganda, the main local language. If needed, a translator was asked to assist. Clinical information was collected by interviewing the next of kin using a standardized questionnaire and by reviewing the medical chart of the deceased. After informed consent was obtained, the autopsy was performed within 12 hours. The body was embalmed free of charge afterwards. Patients that died without an available adult relative were excluded from the study.

### Clinical diagnosis

The doctor on the ward was asked for the clinical cause of death and any contributory condition(s). Afterwards the study team reviewed all medical charts to collect diagnostic evidence. Four groups of clinical diagnoses were defined:

1. confirmed: microbiologic or histologic evidence supporting the diagnosis (e.g. smear (+) tuberculosis (TB))
2. highly suspected: radiologic findings suggestive of a specific disease (e.g. an x-ray or abdominal ultrasound suggestive for TB), a highly suggestive clinical presentation (e.g. cutaneous lesions suggestive of KS) or patients referred from elsewhere while on disease-specific treatment.
3. considered: suspicion based on the clinical presentation with negative and/or unavailable results from investigations or without additional investigations performed at the time of death.
4. not considered: not mentioned by the ward doctor or in the medical chart.

HIV status was abstracted from the medical chart. For those unaware of their serological status on admission, provider-initiated, free, opt-out HIV testing had been offered according to the hospital guidelines. The algorithm for rapid testing involved 3 sequential HIV tests: Determine TM (Abbot Laboratories by Abbot Japan CO. LTD, Minato-KU, Tokyo, Japan), HIV 1/2 Stat-Pak (Chembio Diagnostics Systems, 3661 Horseblock Road, Med Ford, New York, 11763, USA) and Unigold TM (Trinity Biotech PLC, IDA Business Park, Bray, Cowicklow, Ireland).

### Autopsy and histological examination

After informed consent was obtained, a complete autopsy examination was performed, eviscerating all organs including the brain. Standard tissue sections were taken from every organ and from any macroscopically detected lesion. All samples were fixed in 10% formalin solution. Fixed tissue samples were processed for routine hematoxylin and eosin stain (H&E) following standard protocols.

The H&E stained slides were examined by light microscopy by three experienced pathologists (R. Lukande, E. Van Marck and A. Nelson). When indicated, special stains for organisms were done including Ziehl-Neelsen (ZN), Grocott-Methenamine Silver, Brown-Hopps Gram, Periodic-Acid Schiff and mucicarmine. Acid fast bacilli seen after ZN staining were classified as *mycobacterium tuberculosis*, taking into account possible errors due to mycobacterial infections caused by other mycobacteria. When indicated we confirmed the diagnosis of Kaposi's sarcoma and cytomegalovirus infection by immunohistochemistry using commercially available mouse monoclonal antibodies; LANA1 for HHV8, Cell Marque (reference number 265M-18) and CMV antibody, clone DDG9/CCH2, Ventana (reference number 760-2638). The cause of death and contributing findings were formulated based on the clinical information combined with the macro- and microscopic post-mortem findings.

### Ethical consideration

The study received ethical approval from the Makerere University Research and Ethics Committee, the Mulago Internal Review Board and the Infectious Diseases Institute Scientific Review Committee. The study received final approval and registration by the Uganda National Council of Science and Technology (ADM 154/212/01).

### Statistical methods

Data were analyzed using STATA version 11.0 (Stat Corp., College Station, TX, Texas, USA). Data are expressed as mean with a 95% confidence interval (95% CI) or as median with a range.

## Results

**Figure 1:**
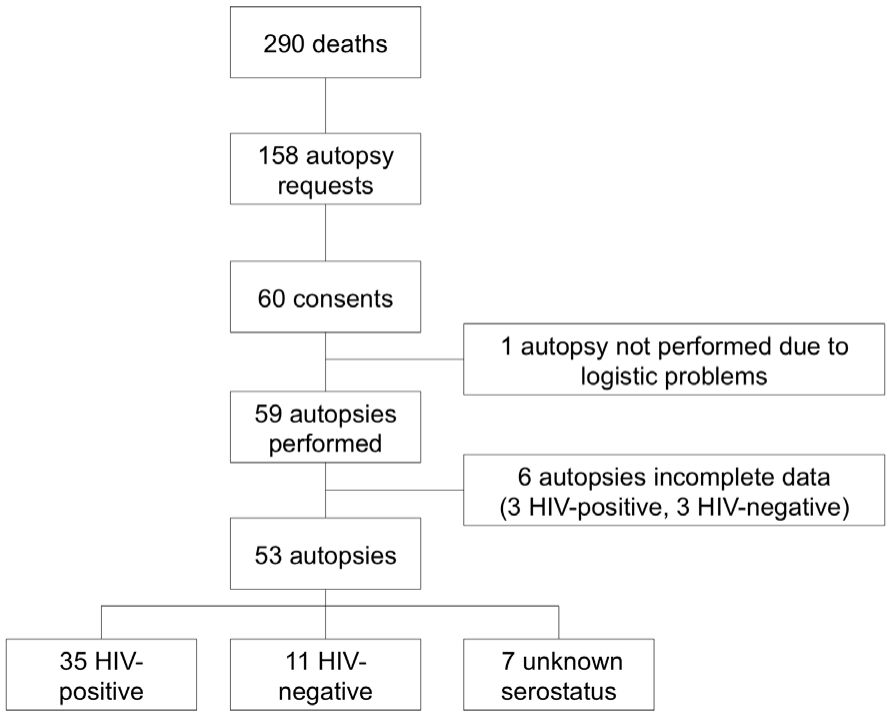
Study flow diagram.

During the 5 month-study period 290 patients died on the study ward. An autopsy was requested in 158 (54%) and performed in 59 (37%) of them. In this paper we present the autopsy data of 53 patients; 66% HIV-positive, 21% HIV-negative and 13% patients with an unknown serological status (table 1, figure 1). Details of the entire study cohort including those that did not consent to autopsy were previously described [12]. Six patients (3 HIV-positive patients, none on ART) were left out of the analysis due to incomplete postmortem data.

**Table 1:** Patient characteristics (n = 53).

	HIV-positives (n = 35)	HIV-negatives (n = 11)	Unknown serological status (n = 7)
Mean age in years (95% CI)	38 (35–42)	41 (32–49)	45 (24–66)
Sex (% male)	48	64	71
Mean Karnofsky score (95% CI)	38 (30–46)	31 (25–37)	28 (12–45)
Average admission duration	7.4 days	5.8 days	2.0 days
Percentage admitted on gastroenterology ward	14%	73%	71%

On admission 16 (30%) of the 53 patients were unaware of their HIV serological status. Six (38%) tested HIV-positive and 4 (25%) HIV-negative during hospitalization. One patient tested HIV-negative according to the medical chart, however according to the relatives he was HIV-positive. He was classified as a patient with unknown serological status for the analysis. All patients, except one, with an unknown HIV serological status died within 48 hours after admission. The median duration of hospitalization before death was 5 days (range <1–30 days). According to relatives, 61% of patients had symptoms of the current illness for more than one month. Ninety-one percent of patients had sought medical care prior to admission and 55% had done so 30 days or more before admission. Of the 16 patients with unknown HIV serological status on admission, 88% had sought medical care prior to admission.

A CD4 T-cell count was available for 13 (36%) HIV-positive patients. Their mean CD4 T-cell count was 50 cells/mm^3^ (95% CI 14–87). Ten (27%) patients were reported to be on ART before they died. Six patients had been on ART for less than 2 months; the other 4 were on ART for 8, 12, 36 and 48 months respectively. All were on a NNRTI-based regimen. No patient was started on ART during the admission in Mulago Hospital. For 6 of the patients on ART, CD4 T-cell counts were known. In one patient on ART for 12 months, the CD4 T-cell count was only 29 cells/mm^3^. In another patient on ART for 36 months the CD4 T-cell count was only 26 cells/mm^3^. In 4 patients who started ART less than 2 months before admission, the CD4 T-cell count pre-ART ranged from 2 to 82 cells/mm^3^. No pre-death ascertainment of HIV viral loads was available to assess non-adherence or viral failure. Of the 30 patients aware of their HIV-positive serological status on admission, 93% were on cotrimoxazole prophylactic treatment.

### Autopsy findings

**Figure 2:**
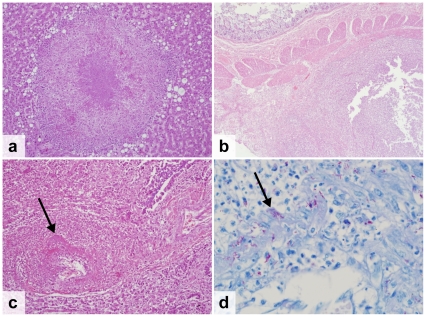
Spectrum of host response to mycobacterial infection in HIV-infected patients. a. Miliary nodule in the liver with a small granuloma in a patient with disseminated disease (H&E stain) b. Abscess in the submucosal lymph nodes of the small bowel (H&E stain) c–d. TB vasculitis in a lung vessel. The arrow indicates acid-fast bacilli in the pulmonary blood vessels (H&E stain, 20× and ZN stain, 100×).

**Figure 3:**
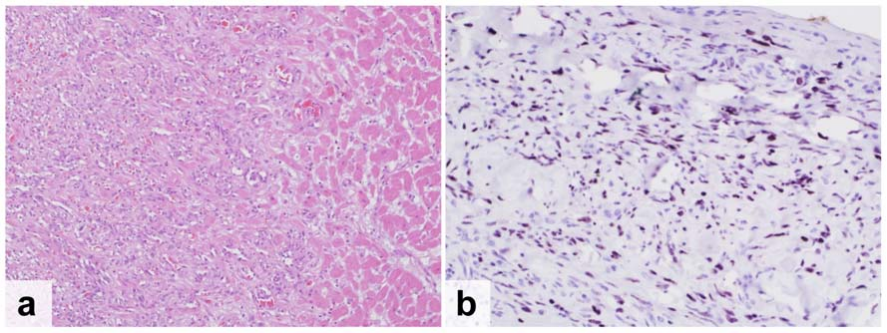
Kaposi's Sarcoma. Disseminated Kaposi's Sarcoma (KS) in a patient who was on lamivudine, zidovudine and nevirapine for 8 months and was treated for KS for 3 months. a. Note vascular proliferation of KS invading cardiac muscle (H&E) b. Kaposi's associated Herpes virus detected in the pericardium (immunophenotyping using LANA1).

**Figure 4:**
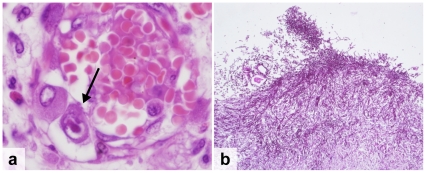
Opportunistic infections. a. Cytomegalovirus infection showing an “owl-eye” nuclear inclusion in an endothelial cell (H&E stain, arrow) b. Esophageal candidiasis showing yeast and pseudohyphae (PAS stain).

**HIV-positive patients:** Overall, 83% of the HIV-positive patients died as a result of infectious diseases (table 2). The leading cause of death was tuberculosis (TB), which accounted for 37% of all deaths (n = 13) (figure 2). Another 9% of patients had TB infection that was not considered their cause of death (n = 3). In all TB patients, the disease was disseminated beyond the lung parenchyma. The most frequently infected organs were the spleen (81%), liver (69%), lymph nodes (69%) and lungs (56%). The second most common cause of death was a *Cryptococcus neoformans* infection that accounted for 20% of deaths (n = 7). One additional patient had disseminated *Cryptococcus neoformans* infection but died of pulmonary haemorrhage due to disseminated Kaposi's sarcoma (KS). In all but one patient, the cryptococcal infection was located in the meninges (88%). Other organs involved included the spleen (50%), the liver (38%), the lungs (38%), the kidneys (38%) and the lymph nodes (38%). Disseminated KS was the only identified malignancy and caused death in 3 patients (9%) who all had widespread involvement including the gastrointestinal tract (67%), the lungs (67%) and the peri- and myocardium (33%) (figure 3). Other less common causes of death included bacterial meningitis (9%), progressive multifocal leucoencephalopathy (3%), *Pneumocystis jiroveci* pneumonia (PJP) (3%) and disseminated cytomegalovirus infection (figure 4a). The latter was a patient who was tested HIV-positive on admission and who was not on cotrimoxazole prophylaxis. Pneumonia was found in 14% of patients, but considered to be the cause of death in only 6%. Chronic renal damage suggestive of HIV-associated nephropathy (HIVAN) was found in 9%. Dual infections were common and identified in 26% of the patients (table 3, figure 4b).

**Table 2:** Causes of death and contributory findings according to HIV-serological status.

	HIV-positive (n = 35)		HIV-negative (n = 11)		Unknown serological status (n = 7)	
	Cause of death	Contributory finding	Cause of death	Contributory finding	Cause of death	Contributory finding
**Infectious diseases**						
Disseminated TB	13 (36%)	3 (9%)	1 (9%)	-	-	-
*Cryptococcus neoformans* infection	7 (20%)	1 (3%)	-	-	-	-
Bacterial meningitis	3 (9%)	-	-	-	1 (14%)	-
Septicemia	-	-	1 (9%)	-	1 (14%)	-
Bacterial pneumonia	2 (6%)	3 (9%)	-	-	-	-
*Pneumocystis jiroveci* Pneumonia	1(3%)	-	-	-	-	-
Disseminated cytomegalovirus	1(3%)	-	-	-	-	-
Disseminated candidiasis	-	-	-	-	1 (14%)	-
Cerebral abscess	1 (3%)	-	-	-	-	-
Progressive multifocal leucoencephalopathy	1 (3%)	-	-	-	-	-
Malaria	-	-	1(9%)	-	-	-
**Non-infectious diseases**						
Gastrointestinal bleeding	-	-	-	-	2 (34%)*	-
Liver failure with cirrhosis	2 (6%)**	1 (3%)†	3 (27%)††	1 (9%)¶	1 (14%)†	1 (14%)**
Cardiac failure	1 (3%)	-	1 (9%)	-	-	-
Pulmonary thromboembolism	-	-	1 (9%)	-	-	-
Chronic renal disease	-	3 (9%)¶¶	-	-	-	-
Goitre	-	1 (3%)	-	-	-	-
Atherosclerosis	-	1 (3%)	-	-	-	-
Ischemic cardiomyopathy	-	1 (3%)	-	-	-	1 (14%)
Perforated duodenal ulcer	-	-	1 (9%)	-	1 (14%)	-
**Malignancies**						
Kaposi's sarcoma	3 (9%)	-	-	-	-	-
Small cell lung carcinoma	-	-	1 (9%)	-	-	-
Adenocarcinoma of the stomach	-	-	1 (9%)	-	-	-

* 1 secondary to *Ancylostoma duodenale* infection, 1 of unknown cause.

** of unknown origin.

† secondary to *Schistosoma mansoni* infection.

†† 2 alcoholic, 1 of unknown origin.

¶ cardiac liver cirrhosis.

¶¶ HIV associated nephropathy.

**Table 3:** Dual infections among HIV-infected patients.

Immediate cause of death	Contributing pathology
Bacterial meningitis	Hepatic schistosomiasis
Disseminated cryptococcosis	Bacterial pneumonia
Disseminated cryptococcosis	TB in the lymph nodes
Disseminated Kaposi's sarcoma	Disseminated cryptococcosis
Disseminated TB	Chronic pyelonephritis
Disseminated TB	Pneumonia
Disseminated TB	Esophageal candidiasis
Bacterial meningitis	Disseminated TB
Bacterial meningitis	Bacterial pneumonia

**HIV-positive patients on ART:** At least 8 of the 10 patients on ART died of HIV-related conditions; 50% died of disseminated TB, 20% of disseminated *Cryptococcus neoformans* infection and 10% of disseminated KS. A 32-year-old female that started treatment with tenofovir, emtricitabine and efavirenz 6 weeks prior to admission possibly died of ART-induced liver failure. Liver sections showed an eosinophilic infiltration against a background of cirrhosis. She had a concurrent clinical undiagnosed TB infection in the lymph nodes. One patient died of pneumonia with *Pseudomonas aeruginosa*.

**HIV- negative patients:** The leading causes of death in HIV-negative patients were non-infectious and accounted for 73% of all deaths. Only one patient had TB, which was disseminated to liver, spleen, meninges and kidneys. One patient died of decompensated alcoholic liver cirrhosis. Two patients died of variceal bleeding secondary to liver cirrhosis: one of alcoholic origin and the other of unknown origin. One patient died of cardiac failure, but also had secondary chronic liver congestion leading to cirrhosis. A patient that died of pulmonary thromboembolism also had underlying sickle cell disease.

**Patients with unknown HIV serological status:** Non-infectious diseases accounted for 71% of all deaths. Gastrointestinal bleeding was the cause of death in 3 patients: one patient had a variceal bleeding secondary to liver cirrhosis due to disseminated *Schistosoma mansoni* infection, in the others no bleeding focus was identified. An accidental finding was an *Ancylostoma duodenale* in the small gut of one of these two patients. One patient died of a perforated chronic duodenal ulcer. The infectious causes of death were bacterial meningitis and disseminated candidiasis.

### Comparison between clinical diagnoses and autopsy findings

**HIV-positive patients:** Overall, 49 postmortem diagnoses were made. Of these 12% were clinically confirmed, 27% highly suspected, 16% considered and 45% not considered. The 6 confirmed diagnoses were cryptococcal meningitis with a positive Indian ink in 5 patients and disseminated TB with a positive Ziehl-Neelsen (ZN) stain of sputum and liver tissue obtained with a liver biopsy in 1 patient. The 13 highly suspected clinical diagnoses consisted of TB in 8 patients: 3 patients were referred while on anti-TB treatment, one patient had a suggestive chest x-ray, 3 patients a suggestive abdominal ultrasound and one patient had both a suggestive chest x-ray and abdominal ultrasound. The other highly suspected diagnoses were disseminated KS (n = 2), liver failure on the basis of laboratory results (n = 2) and a cerebral abscess on the basis of a computer tomography of the head. The 8 autopsy diagnoses that were clinically considered were bacterial meningitis (n = 3), cryptococcal meningitis (n = 2), disseminated TB (n = 2) and pneumonia (n = 1). No diagnostic work-up had been done except in two patients: one patient with disseminated cryptococal infection had a negative serum cryptococcal antigen and another patient with disseminated TB had 3 negative ZN sputum smears, high protein in cerebrospinal fluid without a ZN performed and a normal abdominal ultrasound.

The 22 diagnoses that were clinically not considered consisted mainly of TB (23%) and bacterial pneumonia (18%) together accounting for 9 unconsidered diagnoses. Of these, 4 patients appeared to have two postmortem diagnoses: TB and liver failure, TB and bacterial meningitis, pneumonia and TB, pneumonia and cryptococcal meningitis. In all 4 patients, the second diagnosis was either highly suspected (n = 2) or considered (n = 2). Three patients diagnosed postmortem with disseminated TB without cerebral or meningeal involvement, presented with central nervous system symptoms. One patient with postmortem pneumonia presented with diarrhea and a CD4 cell count of 1 cell/mm^3^. Another patient with postmortem bilateral bronchopneumonia was clinically highly suspected for TB based on a chest x-ray.

When considering only cause of death, 14% had a confirmed diagnosis, 34% a highly suspected diagnosis, 20% a considered diagnosis and 31% of diagnoses were not considered.

**IRIS:** The ward doctors reported a clinical suspicion of TB-associated immune reconstitution inflammatory syndrome (IRIS) in 4 patients. Three of them started anti-TB treatment 1–4 months and ART 2 weeks–3 months prior to admission. One patient started ART 2 weeks before and anti-TB treatment 1 week after admission. The autopsy revealed disseminated TB in 3 of them and disseminated cryptococcal infection in one. No signs of IRIS (e.g. granuloma formation) were identified in any of the postmortem specimens.

**HIV-negative patients:** Of the 12 autopsy diagnoses, none were clinically confirmed, 17% highly suspected, 42% considered and 42% not considered. TB was highly suspected on the basis of a chest x-ray and liver cirrhosis on the basis of an abdominal ultrasound. The clinically considered diagnoses were all based on the clinical presentation without investigations done. Five diagnoses were clinically not considered: small cell type bronchogenic carcinoma in a patient with a productive cough since 3 months clinically suspected of pulmonary TB, pulmonary embolism in a patient with sickle cell disease presenting with back pain clinically suspected of a sickle cell crisis, ischemic cardiac failure and liver cirrhosis with variceal bleeding and malaria in a 19-year-old male admitted with fever and jaundice diagnosed clinically as hepatic failure of unknown origin. No malaria testing was performed during admission.

**Patients with unknown HIV serological status:** Of the 9 autopsy diagnoses, none were clinically confirmed or highly suspected, 67% considered and 33% not considered. The clinically considered diagnoses were all based on clinical presentation. The following diagnoses were clinically not considered: cardiac failure in an 80-year old female with a second postmortem diagnosis of upper gastro-intestinal bleeding probably of vascular origin and disseminated candidiasis and liver cirrhosis of unknown etiology in a 32 year old male with a discrepant HIV serostatus according to the medical chart and the relatives.

**Figure 5:**
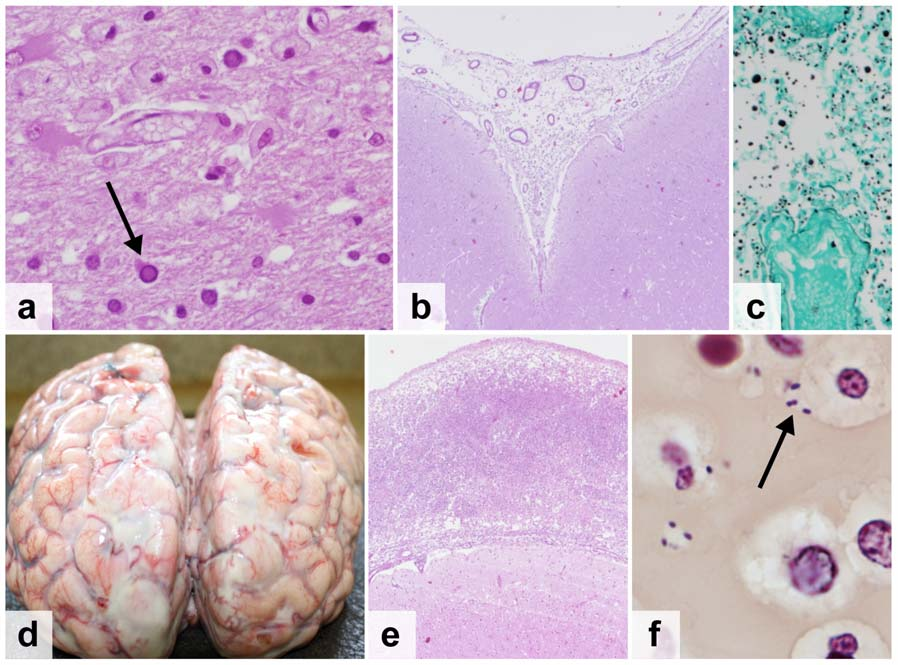
Central nervous system infections. a. Progressive Multifocal Leukoencephalopathy due to JC-virus infection, showing viral inclusion (arrow) and loss of myelin (H&E stain) b–c. Cryptococcal meningitis, showing *Cryptococcus neoformans* with a polysaccharide capsule (H&E stain)(b) and multiple narrow-necked budding yeast (Grocott Methanamine Silver stain) (c) d–f. Streptococcal meningitis, showing the macroscopic image of the brain with edema and purulent exudate (d), the subarachnoid space expanded by neutrophils and fibrin (H&E stain) (e) and Gram positive diplococci (arrow) (Brown-Hopps tissue Gram stain) (f).

**Pathology of the central nervous system:** The brain was eviscerated and sampled in all 53 of the autopsies. In 15 patients (28%), there was evidence of an infectious pathogen. Thirteen of the 15 patients (80%) were HIV infected. Cryptococcal meningitis was present in 6 patients, bacterial meningitis in 4, TB in 2, candidiasis, cerebral abscess and PML each in one patient (figure 5). In all of them, the brain pathology was considered the cause of death except in one patient where cryptococcosis was considered as ‘significant other pathology contributing to death’ in a patient who died of disseminated KS. Brain pathology had been clinically suspected in 14 of the 15 patients (93%).

## Discussion

We found that more than 80% of HIV-positive patients died of infectious diseases with disseminated TB as the most common diagnosis (36%). Furthermore, the spectrum of illness and causes of death were substantially different from those in HIV-negative patients. These findings are similar to those reported in autopsy studies performed in the pre-ART era in sub-Saharan Africa in which infectious diseases caused nearly all deaths with (disseminated) TB as the main cause of death [8], [13], [14], [15].

In our study, the patients on ART died from the same diseases as those not yet on treatment. This is in line with the observational data on early mortality after the start of ART [9], [10]. The patients on ART more than 6 months with a documented CD4 T-cell count had little evidence of immune reconstitution. We concluded that the patients in our cohort were either on ART for too short a time or that they were non-adherent and/or on a failing regimen and therefore showed similar pathology as those not yet on ART. Coverage of cotrimoxazole prophylactic treatment was 93%. Given the small sample size of our study it is not possible to determine the influence of this high cotrimoxazole coverage on disease prevalence or cause of death.

Eighty-eight percent of patients unaware of their HIV serological status had been to a health facility prior to admission. This implies that despite World Health Organization and national guidelines, provider-initiated HIV testing in health care facilities is not yet part of standard medical care in Uganda [16], [17]. By not being tested during an earlier encounter with a health care facility, those that tested HIV-positive on admission missed the opportunity for an earlier therapeutic intervention. Causes of death did not seem to differ between the HIV-positive patients diagnosed on admission and those already aware of their serological status before. This should be considered a reflection of the very poor immune status of the HIV-positive patients already aware of their serological status. Therefore, both earlier diagnostic testing and retention in care should be advocated.

Overall, only 7% of postmortem diagnoses were confirmed and another 21% highly suspected during life. This highlights the difficulty of establishing a certain diagnosis in a resource-constrained setting [18]. This is partly due to the low performance status of many patients (mean Karnofsky scores∼30 on admission) being either too ill or dead before diagnostic testing could be performed or results became available. Moreover, invasive procedures to come to a diagnosis, e.g. LP, were sometimes refused or failed. Some of the diagnostics performed gave false negative results or were interpreted wrongly. This emphasizes the urgent need for rapid, point-of-care diagnostics with high sensitivity. Recent studies evaluating new techniques for diagnosing TB (GeneXpert and urinary lipoarabinomannan) and cryptococcosis (Cryptococcal AntiGene lateral flow immunoassay) show promising results [19], [20], [21]. They are becoming more available but implementation research on the best way to utilize these tests is needed. These tests hopefully will allow us to move from empiric algorithm-based treatment toward diagnosis-based, targeted treatment.

In HIV-positive patients, almost half of postmortem diagnoses were not considered. Main reasons were presentation with dual infections, atypical disease presentation or misinterpretation of the results from additional examination e.g. chest x-ray. Dual diagnoses appear prevalent in HIV-positive hospitalized patients, and high suspicion of a second diagnosis is indicated [14], [15]. Our study shows the value of autopsy in informing empiric algorithms of care. Making clinicians aware of missed diagnosis will lead to an expansion of their knowledge and will inevitably improve patient care.

Because of all the diagnostic difficulties mentioned above, the use of preemptive treatment for highly prevalent conditions like TB or cryptococcal infection should be explored further pending the widespread availability of more sensitive rapid point-of-care diagnostic tests. At this moment, the only preemptive treatment widely used in the Mulago Hospital setting is broad-spectrum antibiotics. More information on the benefits and risks of such treatment strategies is needed.

Our study had several limitations. We enrolled only patients from one ward of a single hospital. This certainly created a selection bias leading to over- and underreporting of some diseases e.g. a patient with focal neurologic complaints due to toxoplasmosis might have been admitted to the neurology ward instead of the infectious diseases ward. Therefore the results should be generalized cautiously. HIV-status was derived from the medical chart and was missing in 11%. In addition, we could not analyze all the patients due to incomplete post mortem data (10%).

In conclusion, our results show that patients continue to die of treatable diseases, especially the HIV-infected patients, and that the clinical diagnosis is often not confirmed prior to death. HIV testing should be performed earlier and at lower level health facilities. As more point-of care diagnostics are developed, empiric algorithms should be traded for diagnosis-based targeted treatment which clinicians alert for multiple pathogens in severely immunosuppressed HIV-infected patients. Finally, our study demonstrates the importance of autopsy examination to ascertain causes of death particularly in settings with limited access to diagnostic testing during life.
